# Clinical research of intraperitoneal implantation of sustained-release 5-fluorouracil in advanced colorectal cancer

**DOI:** 10.1186/s12957-015-0737-9

**Published:** 2015-11-24

**Authors:** Hang Yuan, Bo’an Zheng, Shiliang Tu

**Affiliations:** The Surgical Department of Coloproctology, Zhejiang Provincial People’s Hospital, Hangzhou, 310014 China

**Keywords:** Sustained-release 5-fluorouracil, Colorectal cancer, Safety, Long-term effect

## Abstract

**Background:**

The objective of this study was to investigate the safety and long-term clinical effect of intraperitoneal implantation of sustained-release 5-fluorouracil in patients with advanced colorectal cancer during radical resection.

**Methods:**

A total of 202 patients with advanced colorectal cancer undergoing radical operations were randomly divided into an experimental group (98 cases, intraoperative intraperitoneal implantation of sustained-release 5-fluorouracil 600 mg as local chemotherapy) and a control group (104 cases, without local chemotherapy). The clinical data of the two groups was compared including toxicity, complications, local recurrence rate, distant metastasis, disease-free survival, and 1-, 3-, and 5-year survival rates.

**Results:**

Both groups of patients were followed up for more than 5 years, the longest follow-up time was 7.5 years. Bone marrow suppression, hepato-renal function, postoperative anastomotic leakage, pelvic effusion, incision infection, the incidence of intestinal obstruction, venting time, and hospital stay after operation (days) between two groups had no statistical significant difference. Locoregional recurrence and liver metastasis rate were decreased significantly in experimental group (*P* = 0.04 and 0.04). Extensive peritoneal metastasis and other organ metastasis rates had no significant difference between two groups. In the experimental group, 1-, 3-, and 5-year survival rates were higher than in the control group (95.92 vs 87.50 %, 77.55 vs 64.42 %, and 56.12 vs 40.38 %), which had significant difference. Disease-free survival (DFS) of the experimental group was higher than that of the control group (*χ*^2^ = 5.00, *P* = 0.025).

**Conclusions:**

Intraperitoneal implantation of sustained-release 5-fluorouracil is safe for advanced colorectal cancer during radical resection, which can reduce locoregional recurrence rate and liver metastasis rate. The long-term efficacy was reliable, and long-term survival and disease-free survival rate can be improved.

## Background

The incidence of colorectal cancer (CRC) tops the list among malignant tumors, and its mortality is also very high. Each year, nearly 1,000,000 new cases of CRC are diagnosed and there are 500,000 deaths from CRC [1]. The incidence of CRC in China is lower than in the Western world, but has been increasing in recent years [2]. The primary treatment for CRC is resection of the primary tumor. However, the intra-abdominal recurrence and liver metastasis are the leading causes of deaths in patients with advanced colorectal cancer after a primary operation. The intra-abdominal recurrence is mainly due to exfoliated cells in the peritoneal cavity, retroperitoneal lymph nodes, and metastatic lesions in the peritoneum. Intraperitoneal chemotherapy allows the direct reaction between the drugs and the surface of the peritoneum and the organs in the abdominal cavity; thus, the cancer cells that have dropped from cancer tissues or other small cancer cell clones can be effectively killed, which in turn can prevent the local recurrence of CRC. Many studies reported that intraperitoneal administration of chemotherapeutic agents to eliminate the cancer cells can prevent tumor recurrence. Several agents used in intraperitoneal chemotherapy include cisplatin, 5-FU, hydroxycamptothecin, and slow-release Sinofuan [3–6]. 5-fluorouracil (5-Fu) is the most common and effective chemotherapy drug used to treat CRC. Characterized by long action duration, reaching the maximum drug concentration in targeted lesions, reducing the systemic toxicity, and absorption not through gastrointestinal tract, sustained-release 5-Fu can reduce the drug loss and maximize drug utilization.

In this research, sustained-release 5-Fu was implantated in the peritoneum and para-aortic abdominalis; the results indicated that intraperitoneal implantation of sustained-release 5-Fu is safe for advanced CRC and long-term survival and disease-free survival rate can be improved.

## Methods

### Patients

A total of 202 patients with advanced CRC who had been treated in the Surgical Department of Coloproctology, Zhejiang Provincial People’s Hospital between June 2007 and July 2008 were included in this study. The ages of the patients ranged from 37 to 72 years. The presurgical workup included physical examination, lung CT scan, CT or MRI scan of the abdomen, carcinoembryonic antigen serum level determination, white and red blood cell counts, platelet count, prothrombin time, and determinations of aspartate aminotransferase, alanine aminotransferase, and serum creatinine.

The inclusion criteria were patients with (1) a radical resection and presurgical stages II–IV by colposcopy, ultrasound, CT scan, or MRI; (2) a diagnosis of CRC confirmed by preoperative biopsy and postoperative pathology report; (3) no serious liver and kidney dysfunction; (4) a primary treatment without any other chemotherapy or biologic therapy before the operation; and (5) informed consent.

The exclusion criteria were patients with (1) unresectable distant metastasis, complete intestinal obstruction, severe abdominal adhesion or infection, or emergency operation; and (2) any condition which was unsuitable for chemotherapy.

This study was approved by the ethics committee of Zhejiang Provincial People’s Hospital. All included patients provided informed consent.

### Study design

In operations, when the surgeon confirmed patients can be treated with radical resection, then patients were randomly assigned into two groups by a computer-generated random number table, namely group 1 (98 patients receiving surgery plus adjuvant intraperitoneal chemotherapy) and group 2 (104 patients with surgery plus normal saline).

All patients underwent conventional open operations. No-tumor procedures (including applying incise drape to the incisions, exploring the no-tumor regions prior to exploring the tumor regions, minimizing touching of the tumor, avoiding applying pressure to the tumor, and ligating the vessels around the tumors first) were performed strictly to avoid iatrogenic planting and spread. After the tumor had been removed and reconstruction had been performed (without reconstruction in Miles or Hartmann operation), 2000 to 2500 mL of distilled water was used to rinse the abdominal cavity before it was closed.

### Implanting sustained-release 5-Fu (Sinofuan) for intraperitoneal chemotherapy

The preparation process of the implanted sustained-release 5-Fu (Sinofuan, Chinese Wuhu Zhongren Pharmaceutical Co., Ltd., Batch Number: 20030615) was as follows: first, 5-Fu particles (the diameter of particles within φ0.1~0.5 mm) were put into the coating pan with specially made coating after crushing and sieving (120 meshes) the active ingredients in 5-Fu on the clean workbench; next, medically used polydimethylsiloxane solution was sprayed into the coating pan a few times to form the microcapsules with 5-Fu microsphere as the core and silicone rubber as the envelope, mixed with polydimethylsiloxane solution again after drying and curing for 4 h at 50 °C, pressed into the mold (φ0.8 × 4 mm), and put on the side to solidify for 24 h; at last, it was dried for 4 h under 40 °C and 0.09 MPa and then packaged after inspection and sterilization.

In group 1, before closing the abdomen, a 600-mg sustained-release 5-Fu (Sinofuan) was implanted to the tumor bed, the surface of retroperitoneum and about 2 cm distant to mesenteric vascular artery or abdominal aorta in case of colon cancer or upper rectal cancer, and in case of rectal cancer below the peritoneal reflection, that was implanted to before sacrum, the side wall of pelvic cavity and about 2 cm distant to mesenteric vascular artery or abdominal aorta. While in group 2, no chemotherapeutic agent was implanted in the abdominal cavity.

### Safety evaluation

The effects of the treatments on the recovery, abdominal cavity, and functions of other organs were evaluated according to the common toxicity criteria issued by the National Cancer Institute (NCI-CTCAE v4.0). Complications including fever for more than 3 days, pulmonary infection, and anastomotic leakage were recorded. In addition, other parameters including gas passage time, drug allergy, abdominal drainage volume, peritoneal irritation signs (including abdominal pain and pressing pain), gastrointestinal toxic reaction (including vomit, diarrhea, and hemorrhage), hematologic toxic reaction (including white blood cell count, red blood cell count, and platelet count before and 7 days after the operation), renal toxicity (including elevation of blood urea nitrogen (BUN) and creatinine), and hepatotoxicity (including elevation of enzymes like alanine aminotransferase) were also measured. Adverse events were reported according to the National Cancer Institute common terminology criteria for adverse events (NCI-CTCAE v3.0; http://ctep.cancer.gov).

### Follow-up

Patients were followed up after operation following the National Comprehensive Cancer Network (NCCN) guideline for colon and rectal cancer (version 1.2007; www.nccn.org/patients) by clinical examination, carcinoembryonic antigen level, lung CT scan, and ultrasound of the abdomen every 3 months. Colonoscopy was done 1 year after surgery and then repeated every 1 year in 3 years.

### Statistical analysis

Statistical analysis was performed using IBM SPSS Statistics for Windows version 18 (SPSS Inc., IBM Corporation, USA). Quantitative data was described by means and standard deviations, and analyzed by Student’s unpaired *t* test between groups, whereas qualitative data was described by proportions and analyzed by the Chi-square test. Kaplan-Maier survival analysis was performed. *P* < 0.05 was considered statistically significant.

## Results

### Patients’ characteristics

The patients’ characteristics in groups 1 and 2 are listed in Table 1. No significant difference was found in age, sex, pathological type, clinical stages, and operation method between the two groups (*P* > 0.05). Thus, the background data for all patients was relatively similar. Eighteen cases of stage IV patients in gp1 contained 4 cases with liver metastasis, 10 cases with isolate peritoneal or great omentum metastasis, and 4 cases with other organ metastases. Sixteen cases in gp2 contained 6 cases with liver metastasis, 7 cases with isolate peritoneal or great omentum metastasis, and 3 cases with other organ metastases.

**Table 1:** Baseline clinical characteristics of the 202 patients

Parameter	Group 1	Group 2	*P* value
Age (years)	57.4 ± 3.1	59.3 ± 2.3	
Sex			
Males	50	56	
Females	48	48	0.69
Localization of tumor			
Rectum	58	58	
Colon	40	46	0.62
TNM stages			
II	32	36	
III	48	52	
IV	18	16	0.95
Histological type			
Tubular	58	52	
Papillary	32	43	
Mucinous	8	9	0.40
Differentiation grade			
High	28	33	
Intermediate	48	47	
Low	22	24	0.85
Operation methods			
Right hemicolectomy	24	28	
Left hemicolectomy	10	14	
Dixon operation	38	41	
Miles operation	18	12	
Combined liver metastasis resection	4	6	
Other	4	3	0.75

The patients’ characteristics in group 1 and 2 are listed. No significant difference was found in age, sex, pathological type, clinical stages, and operation method between the two groups (*P* > 0.05). The background data for all patients was relatively similar

### Incidence of postoperative complications

Incidences of postoperative complications in two groups are listed in Table 2. No significant difference was found in postoperative fever, pulmonary infection, incision infection, gas passage time, pelvic effusion, and peritoneal irritation signs between the two groups (*P* > 0.05). Four cases of anastomotic leakage were found in each of the two groups, and no patient required revision surgery. One patient complained of abdominal pain 1 week after operation excluding obstructions in group 1, which were ultimately considered an adverse effect of the drug.

**Table 2:** Postoperative complications and hospitalized days in the two groups

Postoperative complications	Group 1	Group 2	*P* value
Anastomotic leakage	4	4	>0.05
Pulmonary infection	4	5	>0.05
Incision infection	8	10	>0.05
Pelvic effusion	4	6	>0.05
Gas passage time (days)	3.2 ± 0.6	3.3 ± 0.4	>0.05
Hospital stay after operation (days)	14.5 ± 4.2	16.3 ± 3.9	>0.05

Incidences of postoperative complications in two groups are listed. No significant difference was found in postoperative fever, pulmonary infection, incision infection, gas passage time, pelvic effusion, and peritoneal irritation signs between the two groups (*P* > 0.05). Four cases of anastomotic leakage were found in each of the two groups, and no patient required revision surgery

### Toxic effects in the two groups

Toxic effects in two groups are listed in Tables 3 and 4. When assessing hematologic toxicity, it was found that there were no significant differences in parameters like white blood cells (WBC) between the two groups (*P* > 0.05). There was no significant difference in creatinine, alanine aminotransferase (ALT), and gastrointestinal toxic reactions between the two groups (*P* > 0.05). No congestive heart failure was found in either group.

**Table 3:** Toxic and adverse effects in the patients in the two groups (*χ* ^2^ ± s)

Group		WBC (10^9^/L)	ALT (μ/L)	Cr (μmol/L)
Group 1	Preoperative	6.82 + 0.85	27.32 + 12.12	2.31 + 0.42
	Postoperative (one week)	8.95 + 1.23*	38.54 + 10.65**	2.54 + 0.43***
Group 2	Preoperative	5.98 + 0.79	24.75 + 13.68	2.33 + 0.61
	Postoperative (one week)	9.05 + 2.12*	41.04 + 21.23**	2.53 + 0.71***

Toxic effects in two groups are listed. When assessing hematologic toxicity, it was found that there were no significant differences in parameters like white blood cell (WBC) between the two groups (*P* > 0.05). No significant difference in creatinine, alanine aminotransferase (ALT)

*WBC* white blood cell, *ALT* alanine aminotransferase, *Cr* creatinine

No significant differences between the two groups, *P > 0.05, **P > 0.05, ***P > 0.05

**Table 4:** Toxic and adverse effects in patients in the two study groups (according to NCI-CTCAE v3.0)

Parameter	Group 1					Group 2				
	I	II	III	IV	V	I	II	III	IV	V
WBC	4	2	1	0	0	5	1	0	0	0
Platelet count	1	1	0	0	0	2	0	0	0	0
Bleeding	0	0	0	0	0	0	0	0	0	0
Renal toxicity	0	0	0	0	0	0	0	0	0	0
Hepatotoxicity	1	1	0	0	0	2	1	0	0	0
Fever (post-operation >7 days)	3	0	0	0	0	2	1	0	0	0
Infection	9	3	0	0	0	12	2	0	0	0
Diarrhea	0	0	0	0	0	0	0	0	0	0
Vomiting	1	0	0	0	0	1	1	0	0	0
Constipation	0	0	0	0	0	1	0	0	00	0
Cardiac function	0	0	0	0	0	0	0	0	0	0
Pneumonia (X-ray)	4	0	0	0	0	4	1	0	0	0
Peripheral numbness	0	0	0	0	0	0	0	0	0	0
Allergic reaction	0	0	0	0	0	1	0	0	0	0
Abdominal ache (post-operation >7 days)	5	2	0	0	0	5	1	0	0	0

When toxic and adverse events were classified according to the NCI-CTCAE v3.0, there were no significant differences between the two groups. The most common were 7 cases of abdominal ache in group 1 and 6 cases in group 2; 2 cases of hepatotoxicity in group 1 and 3 cases in group 2; and 12 cases of infection in group 1 and 14 cases in group 2. All of the reported events were grade I or II and therefore were mild or moderate

*NCI-CTCAE* National Cancer Institute common terminology criteria for adverse events

### The post-operation treatment

In group 1, 82 patients received systemic chemotherapy for postoperative adjuvant chemotherapy for at least 2 months, including 80 cases of FOLFOX4 chemotherapy and 2 cases of XELOX chemotherapy, while 16 cases had not received adjuvant chemotherapy. In group 2, 84 patients received systemic chemotherapy for postoperative adjuvant chemotherapy at least 2 months, and all received FOLFOX4 chemotherapy. All stage IV patients of two groups received FOLFOX4 chemotherapy.

### Long-term results

**Fig. 1:**
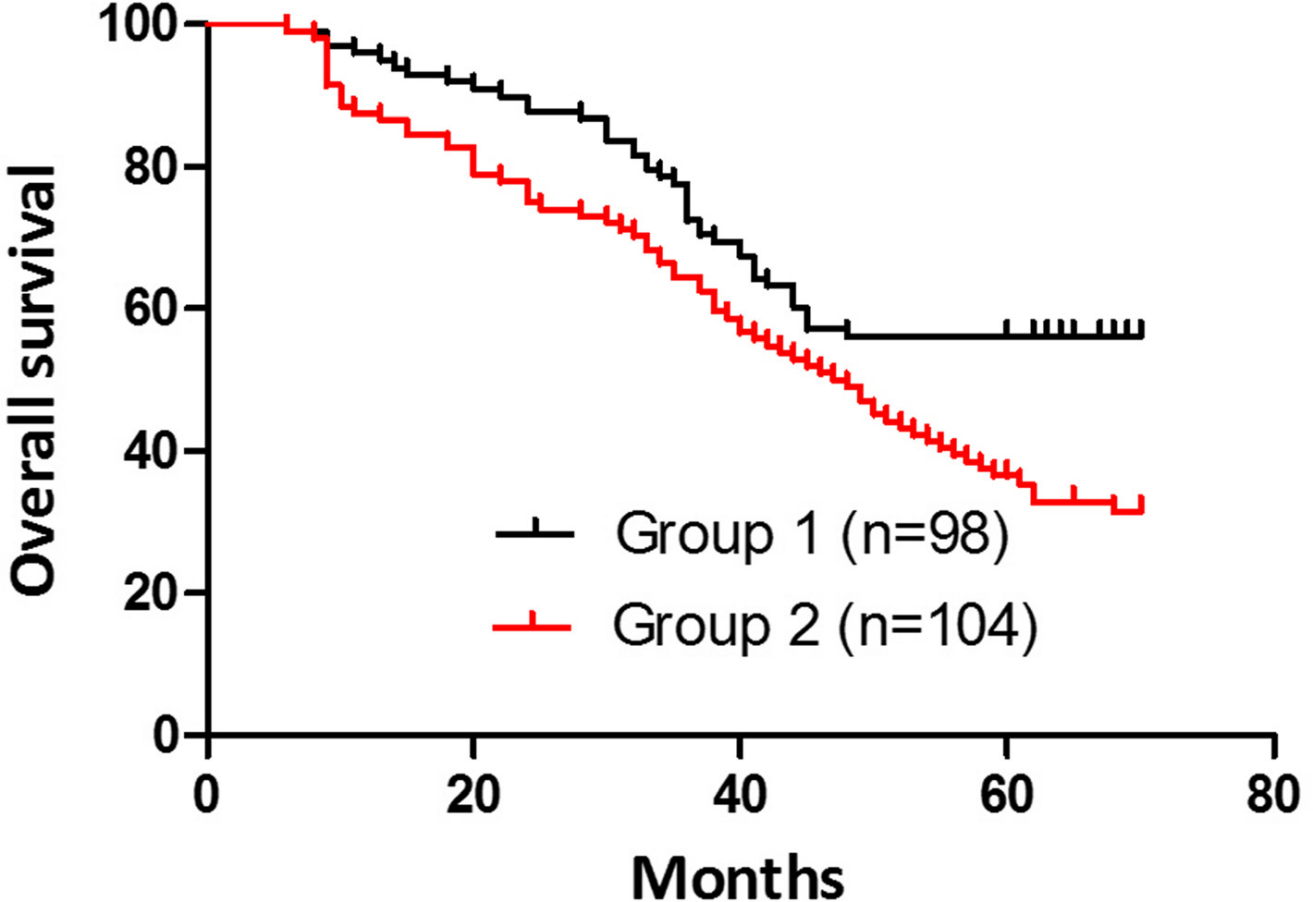
Overall survival curves (group 1, *black line*; group 2, *red line*). The group-specific 5-year overall survival (OS) rates were 56.12 % in group 1 and 40.38 % in group 2 (*p* = 0.03). These differences were statistically significant

**Fig. 2:**
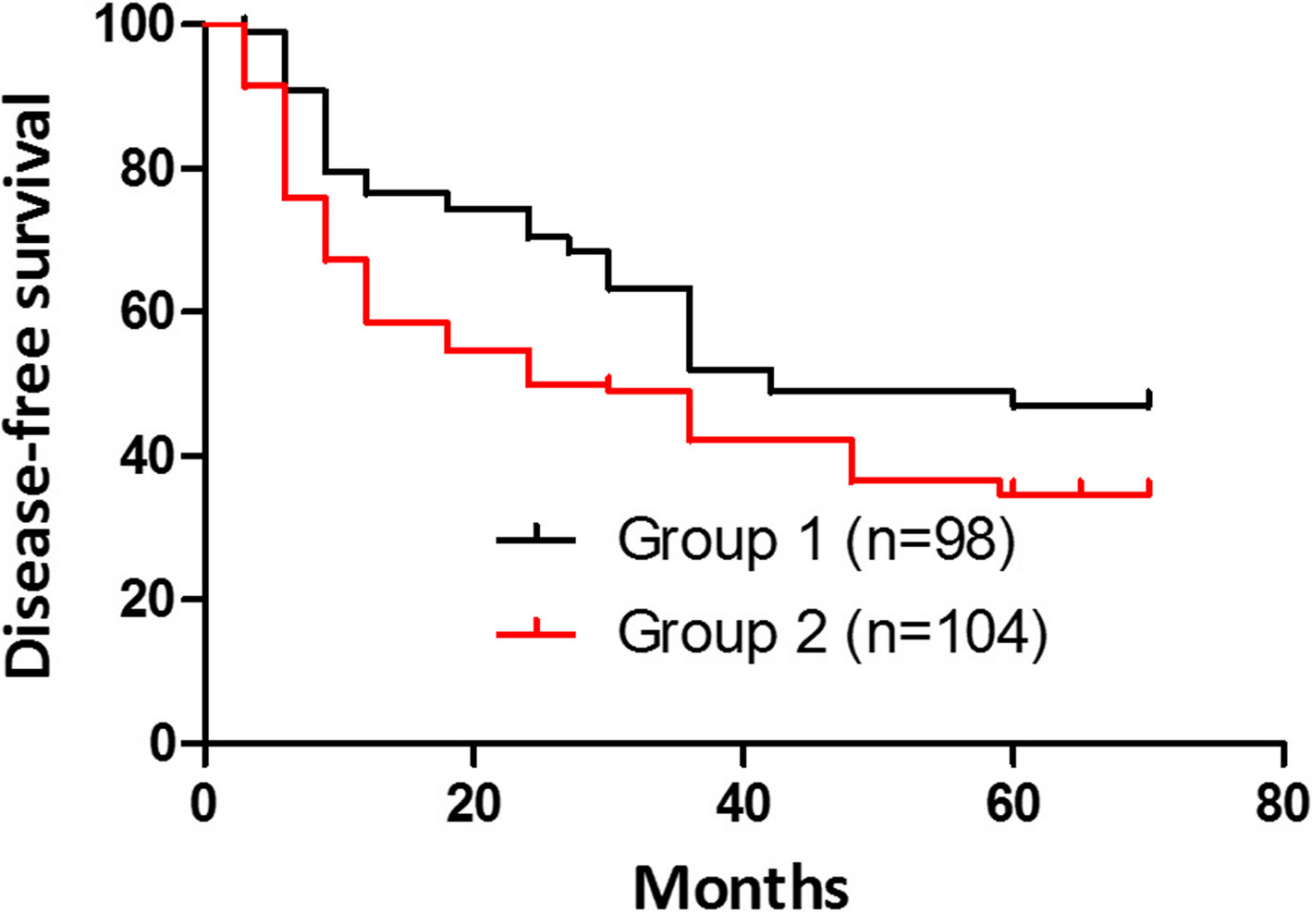
Disease-free survival curves (group 1, *black line*; group 2, *red line*). These differences were statistically significant. Mean disease-free survival (DFS) was 36 months in group 1. The 5-year disease-free survival rates were 48.98 % in group 1 and 34.62 % in group 2 (*P* = 0.04)

The median follow-up time was 70.1 months (range 62–90 months). Four patients had anastomotic recurrence post-operation in group 1 and 13 patients in group 2 (*P* = 0.04). Twenty-eight patients developed to liver metastasis in group 1 and 44 patients in group 2 (*P* = 0.04). Twelve cases had locoregional recurrence in group 1 and 20 cases in group 2 (*P* = 0.04), while there was no significant difference in extensive peritoneal metastasis and other organ metastases. The group-specific 5-year overall survival (OS) rates were 56.12 % in group 1 and 40.38 % in group 2 (*P* = 0.03). These differences were statistically significant (Fig. 1). In groups 1 and 2, 5-year OS rates of stage IV patients were, respectively, 16.7 % (3/18) and 12.5 % (2/16) (*P* = 0.09). Mean disease-free survival (DFS) was 36 months in group 1. The 5-year disease-free survival rates were 48.98 % in group 1 and 34.62 % in group 2 (*P* = 0.04). These differences were statistically significant (Fig. 2). Tumor recurrences (Table 5) were observed in 48 patients (51.02 %) in group 1 and 68 (65.38 %) in group 2.

**Table 5:** Sites of tumor recurrence

Sites of tumor recurrence	Group 1	Group 2	*X* ^2^ value	*P* value
Locoregional recurrence	12/98	20/104	4.04	0.04
Liver metastasis	20/98	30/104	4.15	0.04
Extensive peritoneal metastasis	11/98	8/104	0.64	0.42
Other organ metastases	11/98	6/104	0.84	0.36

Sites of tumor recurrences were observed in 48 patients (51.02 %) in group 1 and in 68 (65.38 %) in group 2

**Fig. 3:**
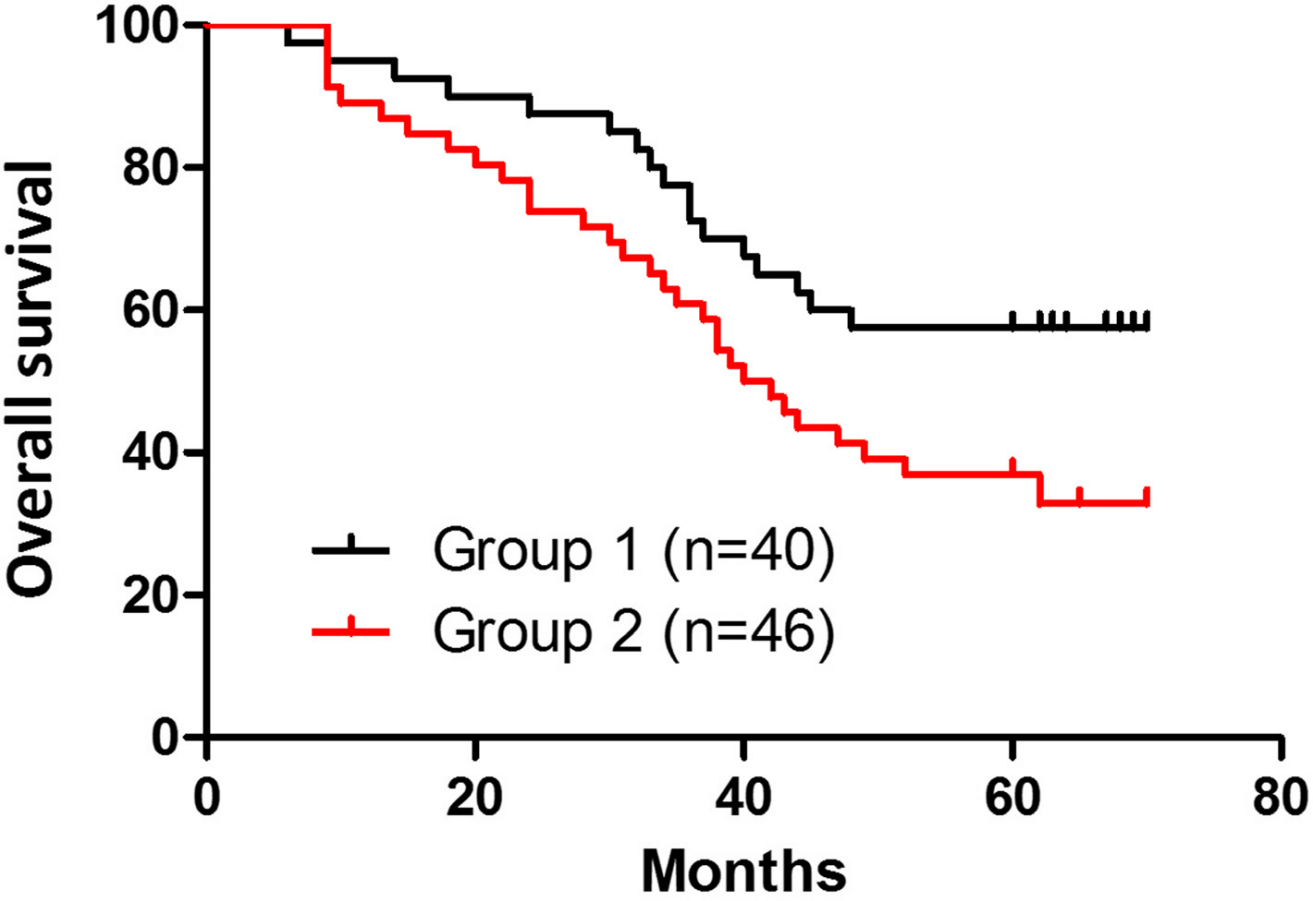
Kaplan-Meier survival analysis (group 1, *black line*; group 2, *red line*). The colonic cancer group-specific 5-year overall survival (OS) rates were 57.5 % in group 1 and 37.0 % in group 2 (*P* = 0.04, significant difference). These differences were statistically significant

**Fig. 4:**
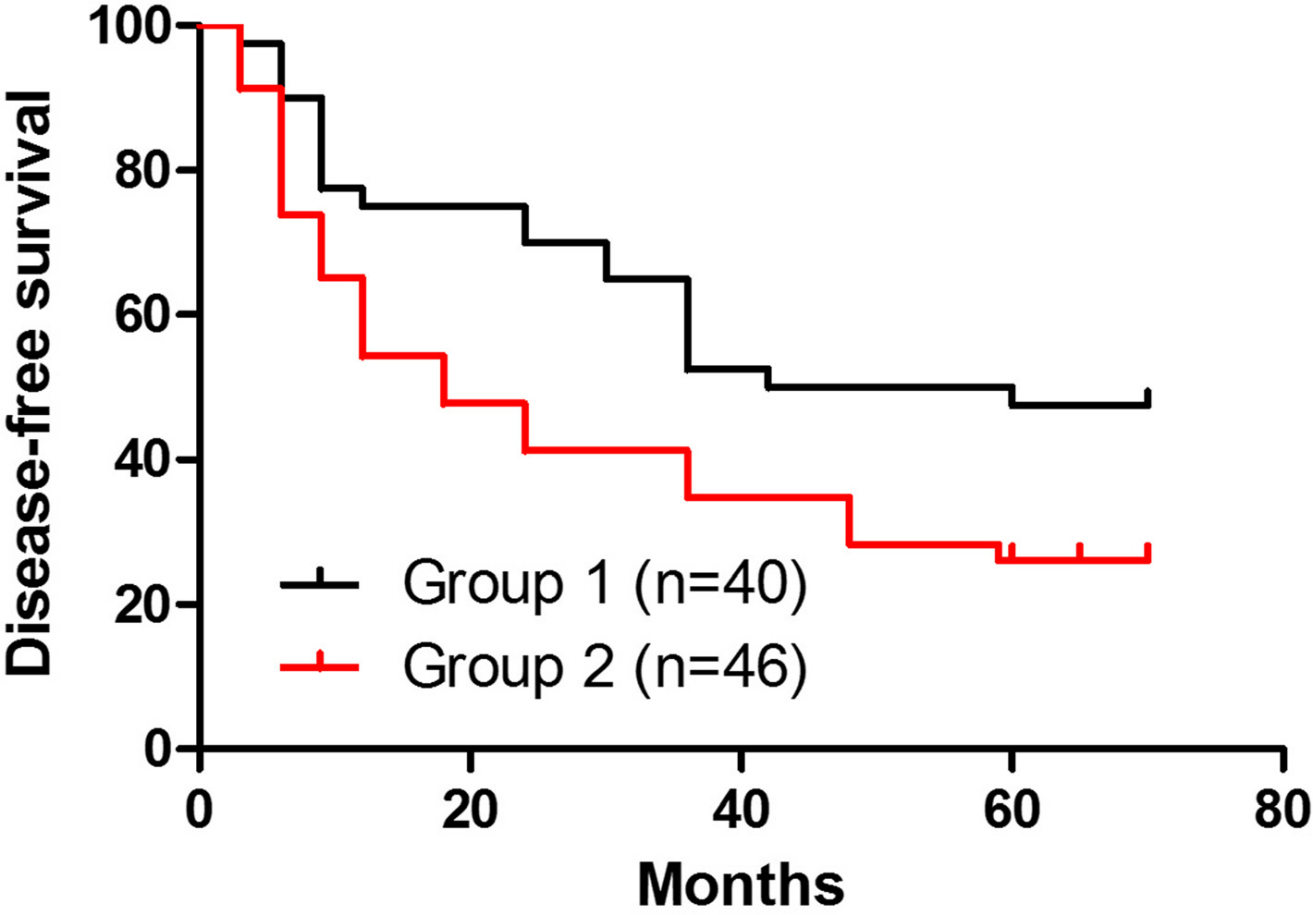
Kaplan-Meier survival analysis. The colonic cancer group disease-free survival curve. Mean disease-free survival (DFS) was 42 months in group 1. The 5-year disease-free survival rates were 47.5 % in group 1 and 26.1 % in group 2 (*P* = 0.02). These differences were statistically significant

**Fig. 5:**
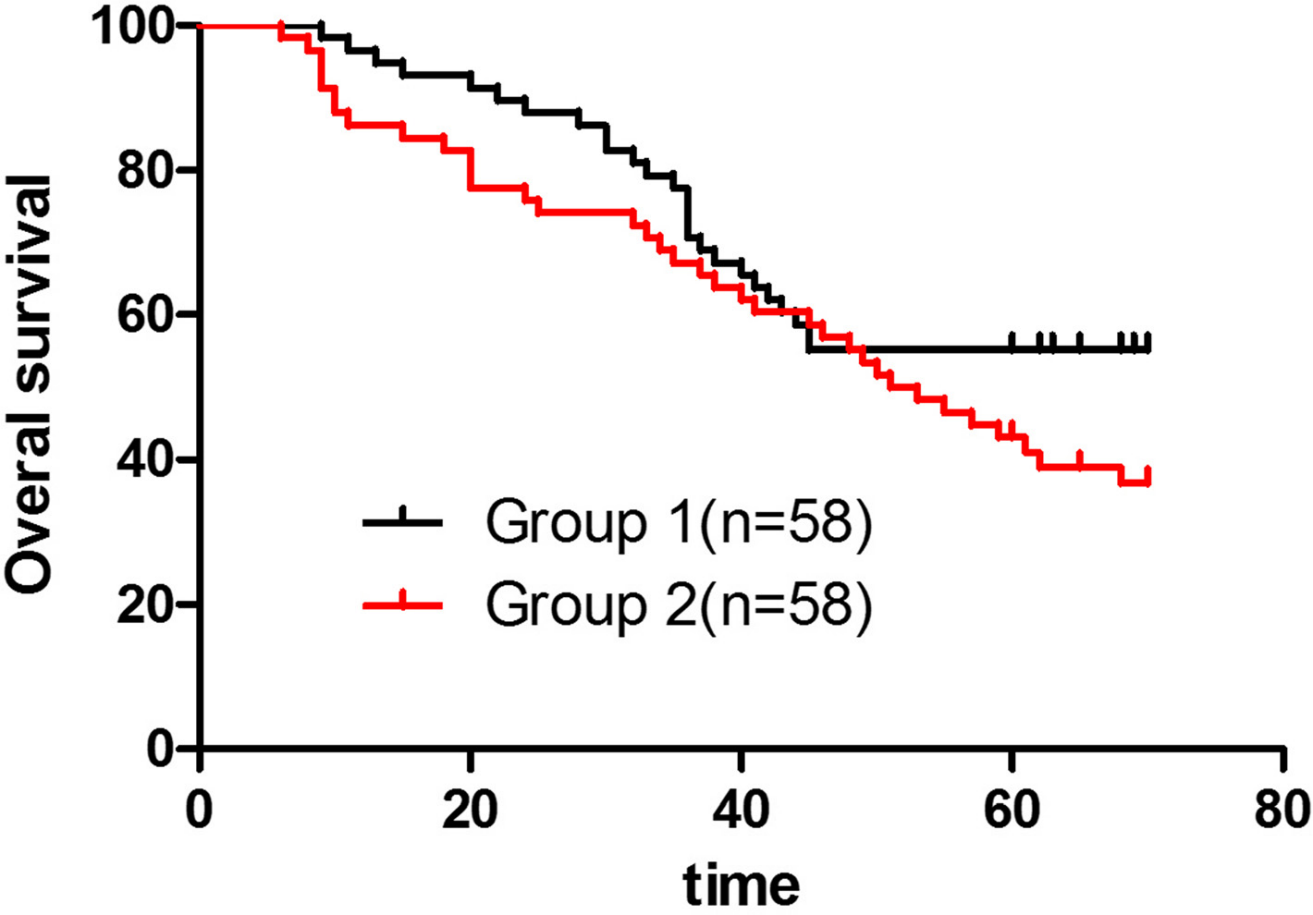
Kaplan-Meier survival analysis. The rectal cancer group overall survival curve. The rectal cancer group-specific 5-year overall survival (OS) rates were 55.2 % in group 1 and 43.1 % in group 2 (*P* = 0.11). These differences weren't statistically significant

**Fig. 6:**
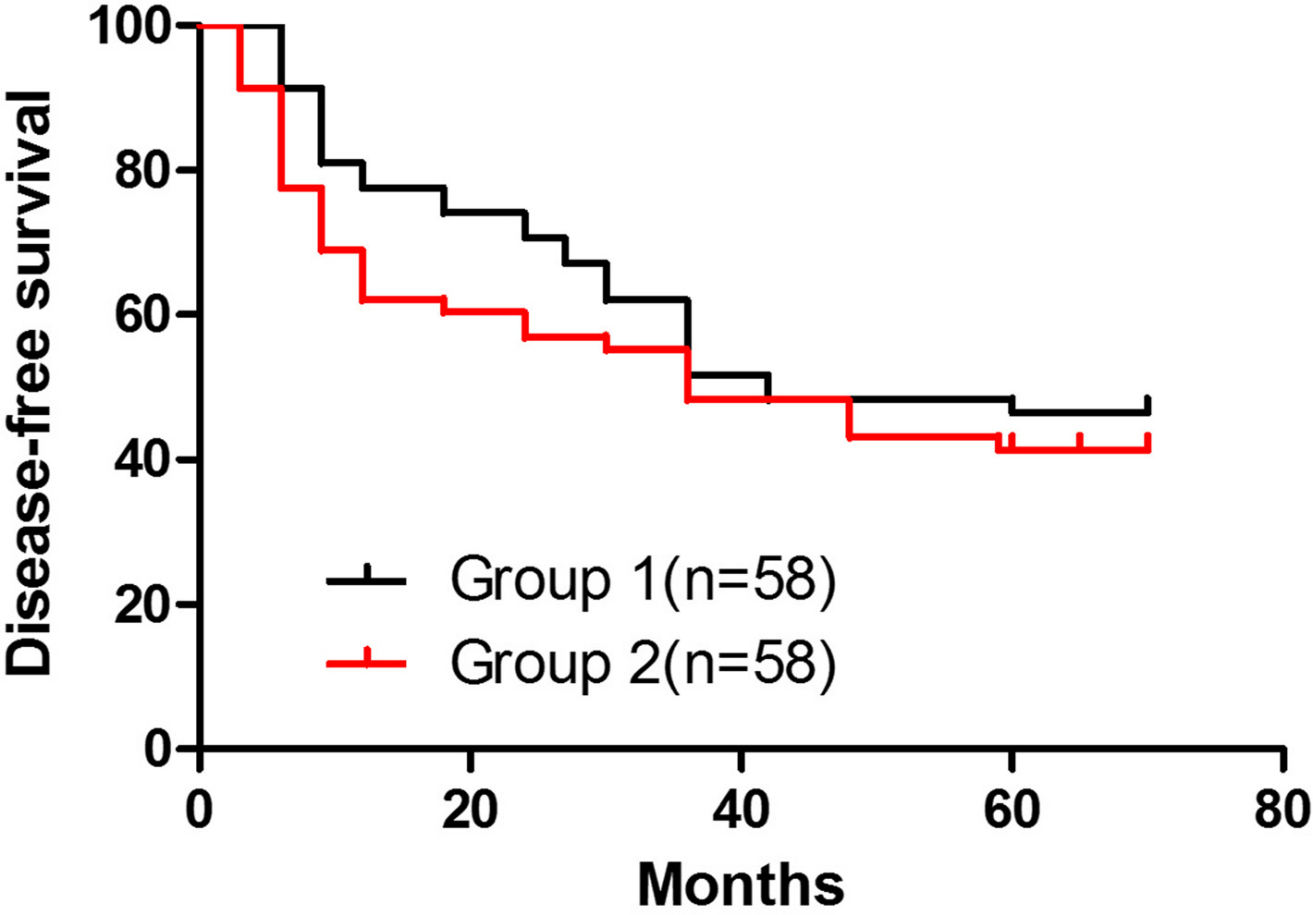
Kaplan-Meier survival analysis. The rectal cancer group disease-free survival curve. Mean disease-free survival (DFS) was 42 months in group 1. The 5-year disease-free survival rates were 46.6 % in group 1 and 41.4 % in group 2 (*P* = 0.36). These differences weren't statistically significant

The colonic cancer group-specific 5-year OS rates were 57.5 % in group 1 and 37.0 % in group 2 (*P* = 0.04). These differences were statistically significant (Fig. 3). Mean DFS was 42 months in group 1. The 5-year disease-free survival rates were 47.5 % in group 1 and 26.1 % in group 2 (*P* = 0.02). These differences were statistically significant (Fig. 4). The rectal cancer group-specific 5-year OS rates were 55.2 % in group 1 and 43.1 % in group 2 (*P* = 0.11). These differences weren't statistically significant (Fig. 5). Mean DFS was 42 months in group 1. The 5-year disease-free survival rates were 46.6 % in group 1 and 41.4 % in group 2 (*P* = 0.36). These differences weren't statistically significant (Fig. 6).

## Discussion

The postoperative recurrence rate of gastrointestinal tumors is very high, even more than 50 %, especially colorectal cancer, which is mainly due to the presence of postoperative peritoneal micrometastasis. The literature has revealed that after primary lesion resection, the residual cancer cells are most sensitive to intraperitoneal chemotherapy in 7 days, and hence, it is the best time to kill residual cancer cells and micrometastases [7]. In 7 days after the surgery, it is improper for the patients to receive the peripheral venous chemotherapy with severe systemic reactions due to poor constitution and incompletely healed incisions. In addition, systemic chemotherapy is not an effective option because of its low drug concentration at the tumor site and short action duration.

This is the only study reported in English that evaluates the safety and long-term effects of intraperitoneal chemotherapy with sustained-release 5-FU (Sinofuan) administered in operation. The implanted sustained-release 5-Fu used in this study is the first long-term sustained-release implant in China, whose active ingredient is 5-Fu. As a new sustained-release implant organically combining high-molecular polymers with good histocompatibility and commonly used drugs recorded in pharmacopeia, it can alter the pharmacokinetics and route of administration, characterized by solid implants, intraperitoneally controlled release, long release time, and locally targeted administration [8]. Peritoneally implanted sustained-release drugs have the following characteristics. (1) Long duration: it can continuously act for several days, months, and even several years. (2) Slowly controlled release: the release of 5-FU is slowly controlled which can reduce systemic toxicity. (3) Target administration: the drug concentrations can reach the highest in the targeted area of lesions, but the total dosage is minimal to reduce the systemic toxicity. (4) Loss reduction: it can effectively make use of drugs to avoid hepatic first-pass effect [9–11]. Therefore, early application of locally intraperitoneal sustained-release chemotherapy can keep a higher drug concentration and longer action duration, consequently achieving the targeted administration and reducing systemic toxicity. Animal experiments showed that drug concentration of intravenous administration in blood after 12 h was reduced to below the tumor inhibition concentration, while the local fluorouracil sustained-release implants after 240 h still remain a valid antitumor drug concentration [12]. A previous study has estimated that chemotherapeutic drugs could eliminate the number of cancer cells ten times when drug concentrations at the tumor/target site are increased at one time point [13]. The research results about sustained-release 5-Fu implantation (Sinofuan) revealed that 10 days after implantation, the drug concentrations in the peritoneum, lymphatic tissue, and portal vein remained relatively high within 5 cm of the implanted site [14].

The killing effect of most chemotherapy drugs depends on time and concentration within a certain range, whereas the efficiency of intraperitoneal chemotherapy drugs depends not only on the drug concentration, but also on the action duration. In conventional chemotherapy, the aqueous solvent of the drug has a shorter action duration in the intraperitoneum, usually 6~8 h. In addition, the depth of chemotherapy drugs penetrating into tumors is limited from the surface. The effect of chemotherapy is greatly affected when the tumor is more than 0.5 cm [15]. However, with a good penetration in the interstitial space and cell membrane, and a certain affinity with tumor tissue, 5-Fu usually enters into the cells through passive diffusion according to a concentration gradient, and can form a higher drug concentration locally and act for a long time [16]. The literature has revealed that 5-Fu wrapped with adjuvant has a good effect in intraperitoneal chemotherapy of gastric cancer [17]. Besides, the efficacy of 5-Fu is positively associated with its action duration [18, 19]. In general, the release time of 5-Fu wrapped with adjuvant does not exceed 1 day, whereas that of sustained-release 5-Fu implantation lasts more than 10 days, which overcomes the disadvantage of conventional chemotherapeutic drugs with a short action duration [20, 21]. Such preparations can also be easily placed at any site of residual tumors during surgery and stably maintain a higher drug concentrations at the administered area for a long time, being conductive to killing the residual tumors not removed during surgery, micrometastasis, and intraperitoneal free cancer cells. In addition, the local administration makes the systemic normal cells have less load; thus, the toxicity of anticancer drugs is kept to a minimum. Therefore, sustained-release 5-Fu implantation is an ideal local intraperitoneal chemotherapy preparation because of sustained-release and targeted dual properties.

Numerous clinical data indicate that the subclinical implanted metastasis formed by invasion of tumors into serosa or implantation of exfoliative cancer cells into the abdominal cavity due to surgery is the leading cause of postoperative local recurrence and liver metastasis in portal system in malignant tumors of the abdominal cavity [22]. A study has found that the detection rate of peritoneal free cancerous cells comes up to 43~55 % in patients with pancreatic cancer [23]. Both surgical procedure and stimulation can increase the exfoliation of cancer cells, and metastasis of peritoneal exfoliated cells will directly lead to the recurrence and death in patients after curative surgery and very low 5-year survival rate in patients with progressive colorectal cancer. The most common malignant tumors in the abdominal cavity easily occur during peritoneal metastasis and liver metastasis, whereas the postoperative conventional treatment including systemic intravenous chemotherapy has a very low efficiency [24]. Currently, intraperitoneal implantation of sustained-release 5-Fu has been widely used in clinics, but its safety and efficacy are still controversial [13, 25].

This study evaluated the safety of sustained-release 5-Fu implantation during surgery. The incidences of postoperative complications had no significant difference. When toxic and adverse events were classified according to the NCI-CTCAE v3.0, there were also no significant differences between the two groups. The relation of the treatment to events reported as signs of poor tolerance is not obvious. Because of sustained-release and targeted dual properties, total dosage is minimal to reduce the systemic toxicity.

Theoretically, the intraperitoneal route combines the effect of intraportal chemotherapy on the liver with a direct effect on the peritoneum and the resection site [26]. The reduction in liver and locoregional recurrence rates observed in our study supports the hypothesis that intraperitoneal chemotherapy combines locoregional and hepatic effects. Because systemic and intraperitoneal routes of administration of adjuvant chemotherapy appear to have different targets and different times of effect, they appear well suited for combined use. About 2 weeks after operations in this study, patients received systemic chemotherapy for postoperative adjuvant chemotherapy according to the NCCN (National Comprehensive Cancer Network) guideline. It is believed that the exfoliation of cancer cells and metastasis of peritoneal exfoliated cells may be killed by Sinofu after surgery, which is supported by the results of this study, in which the group-specific 5-year survival rates and DFS improvement were observed and the difference were statistically significant. It is observed that the 5-year survival rates and DFS have been improved in colonic cancer, not in rectal cancer. Maybe it was due to the different prognosis and guidelines for management. Patients with rectal cancers of stages II or IV received chemoradiotherapy after the operation. Some biomarkers will also have influence on the outcome. Microsatellite instability (MSI) is a marker of prognosis and chemotherapeutic response. The impact of MSI is different between colon and rectal cancers. Because high-frequency MSI (MSI-H) showed a poor response to 5-FU chemotherapy, the treatment regimen may dilute the impact of MSI. This discrepancy may be due to selection bias.

This study has some limitations. The relatively small sample size and single centeredness of the present study limited the statistical power of the study; the difference in adjuvant chemotherapy has an impact on the outcome. Thus, these findings should be interpreted with caution, and further studies with larger sample sizes and multi-center are warranted to validate our findings.

## Conclusions

The findings of the present study showed that there were no significant differences in the anastomotic leakage, postoperative gas passage time, and other complications (including incision infection, pulmonary infection, and hospital stay after operation (days)) between the two groups, suggesting that intraperitoneal implantation of sustained-release 5-Fu (Sinofuan) did not increase the risk of the operation. In addition, no significant difference was found while comparing hematologic toxicity, renal toxicity, hepatotoxicity, and cardiac disease parameters before and at the seventh day after the operation, in each of the two groups. The toxic and adverse events were all graded as I or II according to NCI-CTCAE v3.0 and therefore were mild or moderate. The reduction in anastomotic and pelvic recurrence rates were observed in the present study, which also showed group-specific 5-year survival rates and 5-year DFS improvement, and the difference between the two groups were statistically significant. In this study, using sustained-release 5-Fu implantation during the surgery shows good safety and provides evidences for the prevention and treatment of peritoneal and liver metastases in intraperitoneal malignant tumors and further application of sustained-release 5-Fu in clinics.

## Consent

Written informed consent was obtained from the patient for inclusion into the database and publication of the data.
